# Continuing nursing education: use of observational pain assessment tool for diagnosis and management of pain in critically ill patients following training through a social networking app versus lectures

**DOI:** 10.1186/s12909-020-02159-5

**Published:** 2020-08-03

**Authors:** Kolsoum Deldar, Razieh Froutan, Alireza Sedaghat, Seyed Reza Mazlom

**Affiliations:** 1 School of Paramedicine, Shahroud University of Medical Sciences, Shahroud, Iran; 2 Nursing and Midwifery Care Research Center, Mashhad University of Medical Sciences, Mashhad, Iran; 3 Department of Medical Surgical Nursing, School of Nursing and Midwifery, Mashhad University of Medical Sciences, Mashhad, Iran; 4 Lung Disease Research Center, Faculty of Medicine, Mashhad University of Medicine Sciences, Mashhad, Iran

**Keywords:** Social networking app, Critical-care pain observation tool, Nurse, Education, Intensive care unit, Lectures

## Abstract

**Background:**

Nursing staff training in using observational pain assessment tools is highly important to improve the assessment of pain. The present study was conducted to examine the effect of two different training methods (lectures vs. a social networking app) on the diagnosis and management of pain in mechanically-ventilated patients.

**Methods:**

This quasi-experimental study was conducted on 70 nurses working in two Intensive Care Units (ICU) in Mashhad, Iran. The nurses were trained in the application of observational pain assessment tools by lectures or through a social networking app. Before and after the intervention, the nurses’ performance was evaluated in both groups using a checklist based on Critical-Care Pain Observation Tool (CPOT).

**Results:**

In the pre-intervention phase, the nurses’ performance scores in the domains of pain diagnosis and pain management were not significantly different between the two groups (*P* > 0.05). Following the intervention, the mean score of pain diagnosis was 82 ± 19 in the lecture group and 97 ± 8 in the social networking app group (*P* < 0.01), and the mean pain management scores were 30 ± 17 and 90 ± 18 (*P* < 0.01), respectively.

**Conclusion:**

This study showed that learning through a social networking app led to improved diagnosis and management of pain in mechanically-ventilated patients when compared with lectures. Training through social networking applications can therefore be considered as a feasible instructional method for developing nurses’ pain management skills.

## Background

Pain is a common phenomenon and a major stressor in intubated patients [1–3]. In addition to the primary illness, factors such as endotracheal tube suctioning, chest tube insertion, and certain positions on the bed can induce pain in these patients [4–7]. Despite the advances in theories associated with pain control [8–10], pain is still a major problem in critically-ill patients admitted to intensive care units (ICUs), as 64% of ICU patients complain about pain during their ICU admission [11].

ICU nurses are the largest professional group delivering services in ICUs. Critically-ill patients and their families benefit from the attention of highly trained and skilled personnel. Competence is a multidimensional concept in intensive and critical care nursing and pain assessment and management are fundamental components of clinical nursing practice [12]. The failure to properly assess the pain experienced by critically-ill patients with lower levels of consciousness causes poor pain management [13].

Although the patients’ self-reporting of the presence and severity of pain is the preferred method for the diagnosis and management of pain [14], some patients are unable to verbalize their pain for various reasons, including reduced levels of consciousness or intubation [15]. Non-verbal pain management tools should be used in such patients. Although several pain assessment tools are available to nurses and the use of reliable behavioral scales is highly recommended for detecting pain in intubated patients, these scales are rarely used in practice [12, 16, 17]. One of the underlying causes of this deficiency is that nursing staff are not usually equipped with adequate training and skills and do not follow a routine pain management protocol [18].

Apart from knowing about the physiology of pain and effective pain management, nurses also need to be informed about reliable pain assessment tools and be trained to employ them [19–21]. Nonetheless, the results of previous studies have shown that a large number (approximately 50%) of ICU nurses lack knowledge on the key aspects of pain assessment [9].

The time constraints of nurses and the inherent limitations of traditional training methods necessitate the use of other methods, such as e-learning, in continuing education in nursing [22]. Nevertheless, these time and space constraints can be overcome and nurses can receive information on the latest scientific discoveries [23]. The incorporation of social networking apps (SNA) into the training process constitutes a new educational method based on information and communication technology. These applications can cause a dramatic transformation in the practice of education, as they facilitate instructor-learner interaction and cooperation [24].

Despite the high prevalence of pain among mechanically-ventilated patients, there is a lack of effective pain control in these patients, maybe due to insufficient staff training and skills [18, 25–27]. We, therefore decided to provide ICU nurses with the necessary knowledge and skills to use observational pain assessment tools and evaluate the outcomes. The method of training applied included two-way interaction through a SNA in the first group and lectures for the second one. The present study thus sought to evaluate the effect of these two methods on the pain diagnosis and management in mechanically-ventilated patients.

## Methods

### Study type and sample size

This quasi-experimental study was conducted on 70 nurses working in two ICUs of Imam Reza and Ghaem hospitals (the largest hospitals in the Northeast of Iran) affiliated to Mashhad University of Medical Sciences in 2018. The sample size was calculated by a pilot study conducted on 20 people (*n* = 10 per group) and using the formula for the comparison of two means for pain diagnosis and management with a significance level set at 0.05 and power at 0.8. Based on this calculation, the sample size for the main study was determined as 26 per group; however, to take account of potential sample dropout, 35 nurses were allocated to each group.

### Inclusion and exclusion criteria

The inclusion criteria consisted of a minimum of 1 year of experience as an ICU nurse, submitting informed written consent to participate in the study, and no history of participation in Critical-Care Pain Observation Tool (CPOT) training programs. The exclusion criteria included unwillingness to continue participation, transfer to other parts of the hospital, taking a leave for longer than 2 weeks, and failure to attend the training sessions provided.

### Sampling method

Initially, one of the two teaching hospitals was randomly selected with a coin toss as the intervention site (A). The other hospital was selected as the control site (B). Each hospital made up a single group in order to prevent information dissemination and contamination between the two groups. Then, 35 nurses were selected from each hospital from their eligible ICU staff and were assigned to their corresponding group.

### Instruments

The CPOT is mainly concerned with the behavioral indicators of pain in patients unable to self-report pain. This tool measures pain with four indicators, including facial expressions, body movements, muscle tonicity, and ventilator resistance, in critically-ill adult patients under mechanical ventilation and has a minimum score of zero and a maximum of eight. This tool was validated in 2006 by Gelinas et al. [28] and its validity and reliability were confirmed in Iran by Rafiei et al. in 2015 [29].

The tool has been developed for assessment purposes only and therefore lacks items on nursing interventions after pain diagnosis. Nonetheless, since the present study intended to assess the effect of different training methods on nurses’ performance, a new CPOT-based tool had to be designed that would detect nurses’ performance in correctly identifying the presence of pain and its severity (Outcome: Pain diagnosis) and subsequently carry out pain-related interventions (Outcome: Pain management). Valid content was therefore developed for the intended items through a literature review and then were revised using a two-round modified Delphi technique [30] to establish expert consensus by gathering their opinions [31].

#### Round 1

Fourteen items which were extracted through literature review, were presented in an expert panel. The topics of the added items included: Notifying the physician about the presence of pain, documenting the presence and severity of pain in the patient’s medical record, implementing pain-relief interventions, documenting the interventions in the patient’s medical record, and assessing the outcomes of the implemented interventions. Three anesthesiologists, four qualified nurses, and three statisticians were asked to rate the clinical relevance of the items on a 5-point Likert scale (“extremely relevant”, “relevant”, “don’t know”, “probably not relevant” and “definitely not relevant”). Also, they were invited to add any additional relevant items if needed. Then the percentage of agreements were calculated. Items with acceptable agreement and consensus (predefined level was considered as 75%) were added to the final list.

#### Round 2

The revised checklist with five remaining items, was sent back to the same experts via email to be commented on (if needed) and finalized (Table 1).

**Table 1:** Nurse’ performance checklist (based on CPOT)

A. Pain Diagnosis		Yes / No
Nurse can detect the pain indicators from facial expressions of patient, correctly. For example: presence of frowning, brow lowering, orbit tightening and levator contraction or any other change (e.g. opening eyes or tearing during nociceptive procedures).		
Nurse can detect the pain indicators from body movements of patient, correctly. For example: Slow, cautious movements, touching or rubbing the pain site, seeking attention through movements, pulling tube, attempting to sit up, moving limbs/thrashing, not following commands, striking at staff, trying to climb out of bed		
Nurse can detect the pain indicators of patient’s compliance with the ventilator, correctly. For example: coughing, blocking ventilation, frequently activated alarms.		
Nurse can detect the pain indicators from muscle tension of patient, correctly. For example: Strong resistance to passive movements or incapacity to complete them.		
Total Score:	Mean Score:	Corrected Score (Mean %):
**B. Pain Management**		
Nurse notifies the presence of pain and its severity to the physician, immediately.		
Nurse documents the presence and severity of pain in the patient’s medical record.		
Nurse implements pain-relief interventions.		
Nurse documents the interventions in the patient’s medical record.		
Nurse assesses the outcomes of the implemented interventions.		
Total Score:	Mean Score:	Corrected Score (Mean %):

#### Scoring method

The correct items scored one point and the incorrect ones scored zero. The total score was calculated as the average of the scores multiplied by 100 to yield a score out of 100.

### Validity and reliability

#### The face validity

The face validity of the observational pain management checklist was evaluated both qualitatively and quantitatively. For evaluating the qualitative face validity, ten physicians and nurses working in ICUs were invited to comment on the difficulty, relevance, and clarity of each of the items. For assessing the quantitative face validity, the same ten personnel were asked to rate the importance of the items on a five-point Likert scale (1: not important, 2: relatively important, 3: moderately important, 4: fairly important, and 5: completely important). For calculating the item impact score, the relative frequency of the physicians and nurses who gave a score of 4 or 5 to that item was multiplied by the mean importance score of the item. An impact score greater than 1.5 was taken to be appropriate [32].

#### The content validity

The Content Validity Ratio (CVR) and Content Validity Index (CVI) were also calculated in this step. For calculating the CVR, ten experts in instrument development with experience of working in ICUs were asked to score each item on a three-point scale (‘necessary’, ‘useful but not necessary’, and ‘unnecessary’). Then, based on Lawshe’s table, items with CVR values of 0.62 or higher were selected [33]. For calculating the CVI, the same ten experts were invited to rate the relevance of each item. For calculating the item-level content validity index (I-CVI), the number of experts who gave a score of 3 or 4 to a particular item was divided by the total number of experts. A CVI value of 0.78 or higher was considered satisfactory [34]. For calculating the scale-level content validity index (S-CVI), the S-CVI average (S-CVI/Ave) technique was used and an S-CVI/Ave value greater than 0.90 indicated a very good content validity.

#### Reliability

The checklist reliability was assessed by measuring the inter-observer agreement. Accordingly, two assessors evaluated the pain assessment performance of ten ICU personnel with regard to ten ventilated patients based on the pain observation checklist, and the checklist reliability was measured by calculating the intragroup correlation coefficient. The test-retest ICC was found as 0.8, indicating the good consistency of the tool.

### Pre-intervention phase

At the beginning of the study, the checklist was used to evaluate the nurses’ performance with regard to ventilator-dependent patients with decreased consciousness in three positions, including the rest position, the changing position, and the suctioning of tracheal secretions. This phase took about 1 month due to the nurses’ shift changes and selecting the eligible patients.

One of the researchers (RF) plus one external evaluator (out of the research team) with more than 20 years of experience in ICUs assessed the nurses’ performance based on the checklist. These evaluators had knowledge and skills in using pain assessment tools, such as CPOT, and were blinded to the nurses’ group allocations.

These two individuals were selected because of their ongoing and accepted presence in ICUs to train para-medical students. They assessed the nurses’ performance in routine tasks without their awareness and without attracting the personnel’s attention so as to avoid influencing their performance. Every time the personnel assessed and recorded a patient’s pain using the CPOT, the evaluators determined the patient’s pain score concurrently using the same tool. They then recorded the pain score recorded by the nurse and the pain score judged by the evaluator and the nurse’s further actions in their checklist.

### Intervention phase

#### SNA group

In this phase, other researchers (AS with two physician assistants) trained the nurses on the application of the CPOT. They created a group on Telegram messenger and sent join requests to the nurses in the intervention group, who then received training by text, photo, video, and audio messages on a daily basis for 2 weeks.

The educational content included explanations about various pain assessment methods, pain self-reporting methods, and pain assessment methods in patients unable to self-report their pain, and also full explanations about the CPOT and its scoring method and full description of necessary interventions after the diagnosis of pain.

If the nurses had any questions about the application of this tool or the educational content, they were encouraged to discuss it in the group. These questions were answered at the earliest opportunity, and other members’ answers were also discussed. Furthermore, the personnel were asked to send a confirmatory message to the group instructor each day after studying the educational material for that day.

#### LBT group

With a prior notice, the nurses in this group were asked to participate in the training sessions held in the conference room of the hospital. One of the authors (AS) gave lectures including PowerPoint presentations on the same material as that in the SNA group. Overall, two 90-min lectures were given once a week to this group.

### Post-intervention phase

Two weeks after the intervention was completed, the nurses’ performance was re-measured in the same domains with the same instrument and by the same evaluators for 2 weeks.

### Data analysis

The statistical analysis of the data was performed in SPSS Statistics 16 (IBM Inc., Chicago, IL, USA). This study used the Shapiro-Wilks test was used to determine the normal distribution of the variables. Also, the Pearson’s Chi-square test for contingency table analysis and the independent T-tests for between group comparisons were used. McNemar’s test was also used to assess the pain documentation rate in each group, before and after the intervention.

## Results

Seventy participants were enrolled in the study. Two participants in the lecture group were excluded due to non-attendance and one in the SNA group due to unwillingness to join the social media network. Table 2 presents participants’ characteristics. The two groups did not differ in any of their basic characteristics before the interventions.

**Table 2:** Characteristics of participants. Shown in the table are the means, the standard deviation (±) or frequencsy n (percent)

Variables	groups		*p* value
	LBT (33)	SNA (34)	
	Mean ± SD	Mean ± SD	
Age (year)	34 ± 6	34 ± 7	*P* = 0.92
Work history (year)	9 ± 5	9 ± 6	*P* = 0.90
Gender	N (%)	N (%)	
Male	9 (27)	5 (15)	*P* = 0.21
Female	24 (73)	29 (85)	
Educational level	N (%)	N (%)	
BsC	30 (91)	34 (100)	*P* = 0.11
MsC	3 (9)	0 (0)	

1. T-test, 2. Chi-square, 3. Exact fisher

*LBT* Lecture-Based Training,

*SNA* Social Networking App,

*BsC* Bachelor of Science,

*MsC* Master of Science

Before the intervention, there was no significant difference between nurses’ performance scores in the domain of pain diagnosis in the SNA and LBT groups (*P* = 0.46). Nonetheless, after the intervention, the nurses in the SNA group, acquired significantly higher scores than LBT nurses (*P* < 0.01; Table 3).

**Table 3:** Nurse performance scores in the domain of pain diagnosis before and after the intervention. Shown in the table are the means, the standard deviation (±) and the confidence interval (95% CI)

Performance scores	LBT group (33)	SNA group (34)	*p*-value
Before	33 ± 34 (21.5, 45)	39 ± 28 (28.5, 49)	0.46
After	82 ± 19 (75, 89)	97 ± 8 (94, 100)	< 0.01

1. T-test

*LBT* Lecture-Based Training,

*SNA* Social Networking App

Similarly, before the intervention, there was no significant difference between nurses’ performance scores in the pain management domain (*P* = 0.59). Nonetheless, after the intervention, the nurses in the SNA group had better scores than in the LBT group (*P* < 0.01; Table 4).

**Table 4:** Nurse performance scores in the domain of pain management before and after the intervention. Shown in the table are the means, the standard deviation (±) and the confidence interval (95% CI)

Performance scores	LBT group (33)	SNA group (34)	*p*-value
Before	20 ± 18.5 (13, 26)	18 ± 11 (14, 22)	0.59
After	29.5 ± 17 (23.5, 36)	90 ± 118 (84, 96)	< 0.01

1. T-test

*LBT* Lecture-Based Training,

*SNA* Social Networking App

Before the intervention, 55% of the nurses in the lecture group and 44% in the SNA group documented the patients’ pain in their medical record (*P* = 0.39), whereas after the intervention, 91 and 100% (*P* = 0.22), respectively, did so. The improvement in pain documentation was significant within both groups (*P* < 0.01, for both groups; McNemar’s test).

## Discussion

The present study was conducted to compare ICU nurses’ training in the application of CPOT pain assessment tools using two methods, including SNA and LBT. According to our results, the two groups were not significantly different regarding the nurses’ performance in ​​pain diagnosis and management before the intervention.

Nonetheless, after the intervention, the nurses’ performance showed a significant improvement in the SNA group compared to the LBT group. The comparison of the nurses’ performance scores in the pain management domain was also indicative of a significant increase in the SNA group compared to the LBT group. Although the mean scores in both indices were higher in the SNA group after the intervention, this effect was reportedly the most statistically significant in the pain management domain.

To the best of the researchers’ knowledge, no similar studies were yet conducted to compare training in CPOT application using both SNA and LBT; rather, most of the studies on the subject had used Social Network Sites (SNSs) as a new technology in health communication and research to teach, learn and enhance educational interactions. This technology was also used to create virtual communities to share knowledge in different domains [35, 36]. Their researchers had argued that such networks provide golden out-of-classroom teaching and learning opportunities [37–42].

Exhibiting desirable behaviors is the ultimate goal of designing and executing training programs [43]. The results of some studies, however, have shown that undergoing training in the pain domain does not always lead to more acceptable behaviors among the personnel [44, 45]. Teaching methods have been introduced as one of the most plausible reasons for this problem [46]. For instance, the content of lessons are not thoroughly discussed in traditional classrooms due to the time limitations and the uneven student-teacher interactions; therefore, learning does not truly take place at higher levels of education [47]. Nonetheless, the application of new methods such as SNSs for learning extends the boundaries of interaction beyond the classroom walls and provides greater learning opportunities due to the members’ increased participation in discussions [48]. This finding was confirmed by the improvement in the nurses’ performance in the SNA group. It seems that since the lecture-based classes were mainly held during the morning hours and inevitably attended by tired and drowsy night-shift nurses, the students have experienced a lower quality of learning and been reluctant to participate in class discussions and ask questions about the educational content. Conversely, the staff in the virtual group, who received the educational content at their convenient time and place, discussed deeper questions in their virtual group. The students’ inquiries and questions influence their intellectual formation under different circumstances. In other words, higher-level cognitive processes will not be stimulated in learners unless thoughtful and profound questions are posed [47, 49].

The proper leadership and management of the group along with the members’ involvement are one of the factors that bring success to social networks. A well-trained and skilled person coupled with energetic teaching assistants can initiate and lead online group discussions. Such group is key to the members’ success in acquiring the necessary skills. In the absence of a leader to organize and direct the members’ discussions, the group will gradually deviate from its original purpose and the members will only exchange pointless and non-educational messages [47]. Accordingly, in the present study, three clinical practitioners were present in the group and initiated purposeful educational discussions step by step in the group and answered the nurses’ questions at the earliest opportunity. These efforts maintained the quality of the messages, increased the educational value of the content, and discouraged irrelevant messages sent within the group.

Finally, adequate and standard training is needed for the accurate assessment of pain using assessment tools, including CPOT, and the improvement of nurses’ performance in pain diagnosis and management [22]. Therefore, apart from the provision of useful and appropriate educational content, more effective teaching methods should also be applied. The results of one meta-analysis study indicated that SNS-based interventions are effective in changing health behaviors [50]. Nonetheless, the application of these networks is associated with certain concerns and limitations. For instance, learning how to use SNS poses a major challenge to some healthcare professionals. Unawareness about the technical aspects of SNSs has also been identified as one of the barriers to their application [51, 52]. In addition, considerable doubt exists over data protection and patient privacy [53]. Furthermore, not all the reviewed studies reported the use of SNSs to be helpful or effective for the participants. For example, some students regarded collaborative and group learning as ineffective teaching methods [54]. Another study also reported participants’ reluctance to use Twitter to enhance their educational experience [37]. Nonetheless, this unwillingness was due to the one-way information flow. The creation of an interactive relationship between the students and the professors and among peers (as our study) can help overcome such challenges.

One of the most notable limitations of the present study was that the nurses’ performance was assessed only for a short period after the training. Although nurses’ knowledge and performance may change over time, this study was incapable of continuing the assessment for a prolonged period due to organizational and financial constraints. Although the intervention and control hospitals were selected randomly, the random selection and allocation of the subjects to the lecture and social network groups was not possible because of the chances of cross-group contamination due to the close and ongoing communication between the subjects. Therefore, the candidates from one hospital were assigned to the control group and those from the other hospital to the intervention group. Furthermore, the evaluators of the nurses’ performance had a relatively continuous presence in these departments as nursing students’ instructors. Consequently, their presence was completely natural for the staff. Nonetheless, attempts were made to evaluate the nurses’ performance confidentially in order to minimize the potential impact of the researchers’ presence on the performances.

## Conclusion

This study showed that learning through a SNA leads to improved diagnosis and management of pain in mechanically-ventilated patients when compared with lectures. Training through social networking applications can therefore be considered as a feasible instructional method for continuing development of nurses’ pain management skills.

## Data Availability

The datasets generated and analyzed during the current study are available from the corresponding author on reasonable request.

## Abbreviations

ICU: Intensive care unit
CPOT: Critical-care pain observation tool
LBT: Lecture-based training
SNS: Social network site
SNA: Social network application
